# Uncovering the Challenges of Rare Diseases: Insights From a Retrospective Cross-Sectional Study in Albania (2005-2022)

**DOI:** 10.7759/cureus.52091

**Published:** 2024-01-11

**Authors:** Adela Perolla, Elsuarta Çalliku, Alma Cili, Tatjana Caja, Polikron Pulluqi, Arben Ivanaj

**Affiliations:** 1 Internal Medicine, Hematology, University Hospital Centre "Mother Teresa", Tirana, ALB

**Keywords:** rare hematologic diseases, incidence, prevalence, screening, rare diseases

## Abstract

Background: Diagnosing and treating rare diseases pose significant challenges within global healthcare systems due to their low prevalence and varying criteria for defining them. In Albania, the absence of a dedicated registry for rare diseases exacerbates these challenges. Recognising this gap, a retrospective cross-sectional study was conducted from January 2005 to December 2022 to analyse the incidence and prevalence of rare haematologic diseases in the country, diagnosed in the Hematology Service at the University Hospital Centre "Mother Teresa," which is the sole diagnostic center for blood diseases in Albania. This study aims to provide insights into the frequency of these diseases within the adult Albanian population and seeks to underscore the critical need for improved data collection and research in this field of healthcare.

Objective: The primary objective of this study is to assess the incidence and prevalence of rare hematologic diseases diagnosed at the Hematology Service of the University Hospital Centre "Mother Teresa" in Albania from January 2005 to December 2022.

Materials and methods: This retrospective cross-sectional study employed a descriptive study design, focusing on the analysis of rare hematologic disease incidence and prevalence. The study was conducted exclusively at the University Hospital Centre "Mother Teresa" in Albania, the primary diagnostic center for blood-related disorders in the country. Data collection spanned a period of 18 years, from January 2005 to December 2022, encompassing patient records. Inclusion criteria encompassed adult patients aged 15 years and older who had received diagnoses of rare hematologic diseases during the specified timeframe, without specific operational definitions applied. Non-probability convenience sampling was used, including all eligible cases identified within the study's timeframe, obviating the need for formal sample size calculation. Data were extracted from the records of the Hematology Service at the University Hospital Centre "Mother Teresa," primarily using medical records containing essential patient information. Data analysis utilised software such as EXCEL 16.0 and SPSS (v. 25.0), applying descriptive statistical methods, including frequencies and percentages, to assess the incidence and prevalence of rare hematologic diseases. The study's findings were summarised and presented in a tabular format to provide a clear and concise overview of the results.

Results: Our study identified 64 cases of rare hematologic diseases among adults. Notably, primary myelofibrosis (MF) exhibited the highest incidence and prevalence rate, followed by Waldenström macroglobulinemia (WM) and Gaucher disease (GD) emerging as the most prevalent diagnoses after MF, with 16 and 10 cases, respectively. Several ultra-rare diseases, such as Fanconi anemia and chronic eosinophilic leukemia, were also detected, indicating a significant disease burden, while diseases such as Factor X deficiency and Niemann-Pick disease type C were exceptionally rare.

Conclusion: Diagnosing and treating rare diseases remain formidable challenges in healthcare systems worldwide. This study underscores the need for enhanced awareness, research, and the pressing need for dedicated registries, collaborative research initiatives, and heightened attention to these conditions to enhance our understanding and management of rare hematological diseases, particularly within the Albanian healthcare context.

## Introduction

Rare diseases, despite their individual rarity, collectively represent a significant public health challenge. Defined variably across the globe, a rare disease in the United States is one that affects fewer than 200,000 individuals [1], whereas in Europe, it is defined as affecting no more than five in 10,000 people [2]. In Japan, it encompasses diseases impacting fewer than 50,000 individuals [3]. This diversity in definitions reflects the complexity and challenges in understanding and managing these conditions, and many countries have introduced diverse legislations, regulations, and policies for orphan drugs in the past two decades, aiming to improve access to orphan drugs [4].

Within Orphanet's repository, there are descriptions available for 6,172 distinct rare diseases (RDs), excluding disease groups and subtypes. These RDs exhibit a varied spectrum of the age of onset, with 81.3% (5,018) providing specific information: 3,510 (69.9%) are exclusive to pediatric-onset, 908 (18.2%) have an onset that encompasses both pediatric and adult populations, and 600 (11.9%) exclusively emerge in adults [5]. Globally, rare diseases affect an estimated 350-475 million people, with nearly 7,100 identified types [6-8]. Rare diseases pose challenges not only to patients but also to healthcare providers and the healthcare system as a whole. The limited availability of knowledge, guidelines, and specialised training in the field of rare diseases complicates the process of diagnosing and effectively managing these conditions. This knowledge gap is particularly evident in the field of rare hematologic diseases, which are often complex to diagnose and treat due to their low incidence and non-specific symptomatology [4,9,10].

Patient registries are considered essential tools in addressing the challenges of rare diseases. They play a pivotal role in amassing an adequate sample size for clinical research, facilitating healthcare planning, and promoting the advancement and assessment of diagnostic and treatment methods [10].

In Albania, the lack of a specialised registry for rare diseases compounds the difficulties associated with these conditions. Acknowledging this deficiency, a retrospective analysis was carried out to examine the occurrence and frequency of rare hematologic diseases diagnosed at the Hematology Service in the University Hospital Centre "Mother Teresa," the sole diagnostic facility in Albania for blood-related disorders. This study endeavours to shed light on the characteristics and prevalence of such diseases among the adult Albanian population, emphasising the pressing necessity for enhanced data gathering and research initiatives.

## Materials and methods

This retrospective cross-sectional study employed a descriptive study design to analyse the incidence and prevalence of rare hematologic diseases diagnosed in the Hematology Service at the University Hospital Centre "Mother Teresa" in Albania. The study was conducted exclusively at the University Hospital Centre "Mother Teresa," which serves as the sole diagnostic center for blood-related disorders in Albania. Data collection for this study spanned from January 2005 to December 2022, encompassing a period of 18 years. Inclusion criteria comprised adult patients aged 15 years and older who had received diagnoses of rare hematologic diseases at the Hematology Service during the specified timeframe (Table 1).

**Table 1:** Inclusion and Exclusion Criteria for the Study on Rare Haematological Diseases in Albania.

Criteria	Inclusion	Exclusion
Age	15 years and older	Under 15 years (pediatrics cases)
Diagnosis	Rare haematological disease (ICD-9)	Other diagnoses not within haematological conditions scope
Admission and Diagnosis Dates	Between January 2005 and December 2022	Not meeting the specified date range
Medical Records Completeness	Complete medical records and clear diagnoses	Incomplete medical records or unclear diagnoses

No specific operational definitions were applied in our study, and a non-probability convenience sampling technique was employed. All eligible cases of rare hematologic diseases diagnosed within the study's timeframe were included in the analysis. The sample size was determined by identifying all relevant cases of rare hematologic diseases meeting the inclusion criteria during the study period. No formal sample size calculation was necessary due to the comprehensive inclusion of all eligible cases. Data were collected from the records and registers of the Hematology Service at the University Hospital Centre "Mother Teresa." Information related to patients diagnosed with rare hematologic diseases, including demographic details and specific disease diagnoses, was extracted from the available medical records. The primary data collection instrument utilised for this study was the medical records and registers maintained by the Hematology Service. Information extracted from these sources included patient demographics, diagnoses, and relevant clinical details. All the data collected during the study were analysed using data analysis software, including EXCEL 16.0 and Statistical Product and Service Solutions (SPSS, v. 25.0) (IBM SPSS Statistics for Windows, Armonk, NY). Descriptive statistical methods, such as frequencies and percentages, were employed to assess the incidence and prevalence of rare hematologic diseases. The findings were summarised and presented in a tabular format to provide a clear overview of the study's results.

## Results

The retrospective cross-sectional study conducted at the Hematology Service in Albania from 2005 to 2022 identified 64 cases of rare haematological diseases within the adult population. The age distribution analysis revealed that the ages at the time of diagnosis ranged from 56.8 to 79.3 years, with an average median age of 69 years.

**Figure 1:**
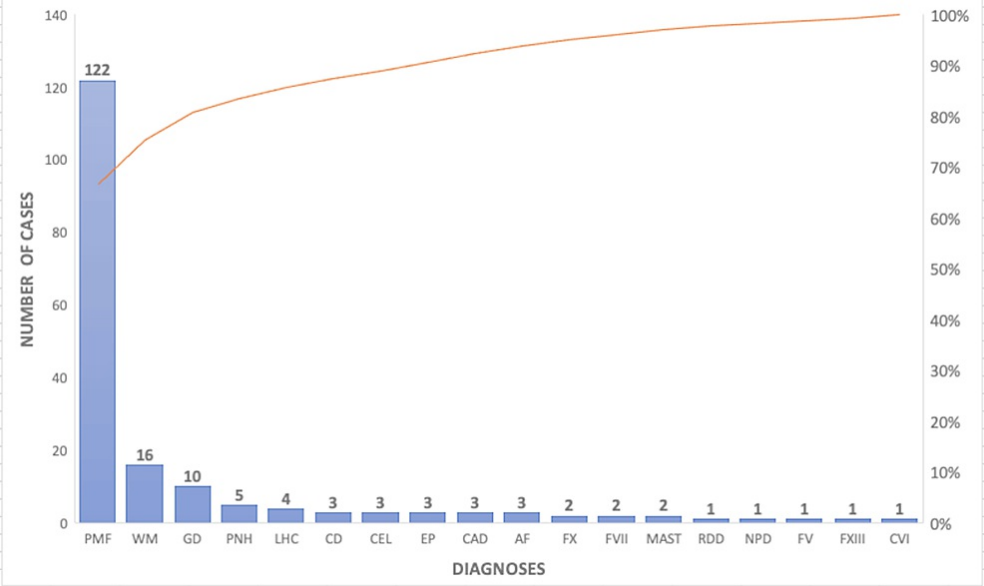
Distribution of Rare Hematological Diseases Diagnosed in Albania (2005-2022). LHC, Langerhans Histiocytosis; FXIII, Factor XIII Deficiency; CVID, Common Variable Immunodeficiency; RDD, Rosai-Dorfman Disease; PMF, Primary Myelofibrosis; GD, Gaucher Disease; AF, Fanconi Anemia; PNH, Paroxysmal Nocturnal Hemoglobinuria; NPD, Niemann-Pick Disease; CEL, Chronic Eosinophilic Leukemia; EPP, Erythropoietic Protoporphyria; CD, Castleman Disease; FX, Factor X Deficiency; WM, Waldenström Macroglobulinemia; CAD, Cold Agglutinin Disease; MAST, Mastocytosis; FX, Factor X Deficiency; FVII, Factor VII Deficiency; FV, Factor V Deficiency The bar chart illustrates the number of cases for each rare hematological disease identified in adult patients within the Albanian healthcare system from 2005 to 2022. Primary myelofibrosis shows the highest number of cases, followed by Waldenström macroglobulinemia, indicating a relatively higher prevalence or better diagnosis rates for these conditions within the studied population.

The bar chart (Figure 1) illustrates the distribution of rare hematologic diseases diagnosed in the adult population of Albania from 2005 to 2022. Primary myelofibrosis (PMF) presented with 122 cases, which is markedly higher than the other conditions in the study, followed by Waldenström macroglobulinemia (WM) with 16 cases and Gaucher disease (GD) with 10 cases. These two diseases represent the most significant proportion of rare haematological diagnoses in the study cohort after PMF.

The chart also shows that other rare diseases have been diagnosed during this period, although in smaller numbers. For example, diseases such as paroxysmal nocturnal hemoglobinuria (PNH), factor X deficiency, and erythropoietic porphyria each account for a few cases, reflecting their lower prevalence in the Albanian population. Notably, ultra-rare diseases such as Niemann-Pick disease and chronic eosinophilic leukemia were diagnosed as well, albeit as single cases.

The analysis of data from the unique diagnostic center for blood diseases in Albania, University Hospital Centre "Mother Teresa" Hematology Service, spanning the years 2005 to 2022, has yielded a detailed assessment of the incidence and prevalence of various rare hematologic diseases within the adult population in the Albanian population (Table 2).

**Table 2:** Summary of the Incidence and Prevalence of Rare Diseases in Albania from 2005 to 2022 in the Adult Population. This table presents a summary of the incidence and prevalence rates for a variety of rare diseases in the Albanian adult population from the years 2005-2022. Each row corresponds to a different disease, detailing: Disease: The name of the rare disease. New Cases in Albania: The total number of new cases recorded in Albania during the study period. Incidence rate (/100,000 adults-year) in Albania: The average annual number of new cases per 100,000 adults, indicating the risk of developing the disease within a year. Prevalence rate (/100,000 adults) in Albania: The total number of existing cases per 100,000 adults as of the end of 2022, reflecting how widespread the disease is.

Disease	New Cases in Albania (2005-2022)	Incidence Rate (/100,000 Adults-Year) in Albania	Prevalence Rate (/100,000 Adults) in Albania
Langerhans Histiocytosis	6	0.013	0.252
PNH	5	0.013	0.252
Gaucher Disease	8	0.018	0.336
Nieman-Pick Disease Type C	1	0.0022	0.042
Factor X Deficiency	2	0.0044	0.084
Factor XIII Deficiency	1	0.0022	0.042
Primary Myelofibrosis	122	0.27	5.130
Fanconi Anemia	3	0.0066	0.126
Cold Agglutinin Disease	3	0.0066	0.126
Mastocytosis	2	0.0044	0.084
Erythropoietic Porphyria	3	0.0066	0.126
Chronic Eosinophilic Leukemia	2	0.0044	0.084
Castleman Disease	3	0.0066	0.126
Waldenström Macroglobulinemia	16	0.035	0.672
Common Variable Immunodeficiency	1	0.0022	0.042

From the data shown in Table 2, it is apparent that PMF presented a significantly higher prevalence rate of 5.130 per 100,000 adults, which is markedly higher than other conditions in the study. Its incidence rate was also the highest at 0.27 per 100,000 adults-year, suggesting either a greater prevalence or an increased ability to diagnose this disease in Albania.

WM was the most frequently diagnosed disease with 16 new cases, after PMF, reflecting the highest prevalence rate of 0.672 per 100,000 adults and an incidence rate of 0.035 per 100,000 adults-year, suggesting that this condition may be more detectable or prevalent within the Albanian population.

GD, with eight new cases diagnosed, had a slightly higher incidence rate of 0.018 and a prevalence rate of 0.336 per 100,000 adults, compared to Langerhans histiocytosis and PNH each accounting for six and five new cases, respectively, both exhibiting an incidence rate of 0.013 and a prevalence rate of 0.252 per 100,000 adults. 

Fanconi anemia, cold agglutinin disease, mastocytosis, erythropoietic porphyria, and Castleman disease each had three cases with an incidence rate of 0.0066 and prevalence rates ranging from 0.084 to 0.126 per 100,000 adults. These conditions, while still rare, showed a small presence in the population.

Niemann-Pick disease type C, factor X deficiency, and factor XIII deficiency were among the diseases with the lowest incidence and prevalence rates, each with a single case or two cases reported, and rates ranging from 0.0022 to 0.084 per 100,000 adults. These figures underscore the rarity of these conditions within the Albanian adult cohort.

Finally, common variable immunodeficiency was identified once, with an incidence and prevalence rate mirroring those of other ultra-rare diseases in the study at 0.0022 and 0.042 per 100,000 adults, respectively.

## Discussion

Our study's findings offer a unique snapshot into the landscape of rare hematological diseases in Albania, a subject with relatively limited literature. The incidence and prevalence rates observed in our cohort from 2005 to 2022 provide a crucial starting point for understanding the burden of these diseases, and valuable insights into the epidemiological landscape of these conditions within the Albanian adult population. Through a comprehensive analysis of incidence and prevalence rates, we have drawn several noteworthy conclusions.

In our study, primary MF exhibited a significantly higher prevalence rate of 5.13 per 100,000 adults compared to the other rare diseases identified in our country. However, when compared to Orphanet's report stating that the prevalence of MF varies between one and nine cases per 100,000 individuals worldwide, this prevalence falls within the globally reported range [11]. We have found an MF incidence of 0.27 per 100,000 adults, significantly lower than the worldwide annual incidence rate of MF reported to be 0.47 per 100,000 person-years and lower than the incidence in Northern Europe, which appeared to be around 0.5 cases per 100,000 person per years ranging from 0.1 to 1 per 100 000 per year [12].

The variation in prevalence and incidence rates of primary MF between our study and Orphanet's global report can be attributed to several factors. Firstly, rare diseases such as MF often exhibit geographic variation, and our study focused on a specific region, where prevalence rates may differ due to genetic and environmental factors. Secondly, differences in data sources, methodology, sample size, and time periods between our study and Orphanet's report can lead to variations in reported rates [11].

Our study found that WM ranks as the second most common rare blood disorder in our population, but less frequent compared to MF, with an incidence of 0.035 cases per 100,000 adults and a prevalence of 0.672 cases per 100,000 adults. In comparison, Orphanet data indicate that WM has an annual incidence rate of one in 260,000 people in the USA [11]. Meanwhile, between 2000 and 2019, the age-standardized incidence rate of WM in the United States was found to be 0.36 per 100,000 people [13] and a prevalence of roughly one in 102,220 in Europe [11]. The prevalence in Albania might suggest an artefact due to the small sample size and demographic differences [14].

The incidence rate of Langerhans cell histiocytosis (LCH) in our study was 0.013 per 100,000 adults per year, with a prevalence rate of 0.252 per 100,000 adults. LCH is a rare condition characterised by the abnormal proliferation of histiocytes, with a diverse range of clinical manifestations that can affect single or multiple organ systems with a prevalence of one to two cases per 100,000 inhabitants [11], and with an occurrence rate of one to two cases per million newborns each year [15]. The incidence of LCH in our study was lower compared to the comprehensive analysis of LCH from the national registry study from England, covering years 2013-2019, and published in 2022 in the British Journal of Hematology, in adults aged 15 years and older, where the incidence rate was at 1.06 per million [16]. Though it can manifest at any age, the condition is more commonly diagnosed in those under 15 years old [16] with an incidence increasing to four to five cases per million annually. For adults, the rate is approximately one to two cases per million per year [17]. Our findings align with global incidence rates, which suggest a rarity but consistent presence of the disease in adult populations [10].

Similarly, the prevalence of PNH in our cohort, with an incidence rate of 0.013 per 100,000 adults-year, reflects the ultra-rare status of this disease [18]. These findings are consistent with previous research, highlighting the scarcity of PNH cases worldwide, with an estimated prevalence of this disease between one and nine cases for every 100,000 individuals [19]. Regarding its global occurrence, PNH has a prevalence of about 15.9 people per million. The yearly incidence rate of PNH on a global scale is approximately five to six persons per one million individuals [20].

Our research identified eight cases of GD, yielding an incidence rate of about 0.018 per 100,000 adults annually and a prevalence rate of 0.336 per 100,000 adults. These rates align with the global estimates for GD, which suggest an incidence range of 0.39-5.80 cases per 100,000 live births and a prevalence of 0.70-1.75 cases per 100,000 people [21]. Such variability in incidence and prevalence, even within the same geographic area, is typical for rare diseases. Despite its relatively low prevalence, GD stands out as one of the more common types of lysosomal storage disorders [21].

In contrast, ultra-rare diseases, such as Niemann-Pick disease type C, factor X deficiency, factor XIII deficiency, and chronic eosinophilic leukemia, demonstrated exceedingly low incidence rates (0.0022 per 100,000 adults-year) and prevalence rates (0.042 per 100,000 adults) in our Albanian cohort. These diseases are exceptionally rare, and our findings align with their status as infrequent clinical entities [22-25]. The occurrence of ultra-rare diseases, such as Niemann-Pick disease and chronic eosinophilic leukemia in our cohort, although in small numbers, is consistent with their reported rarity worldwide, respectively, with the global prevalence of Niemann-Pick disease type C at birth estimated to be between one in 45,000 and one in 286,000 [22] and an incidence rate of chronic eosinophilic leukemia notably low, averaging 0.033 cases per 100,000 person-years over the period from 2001 to 2020 from the SEER database [25]. These diseases represent significant diagnostic challenges and are often underreported, leading to a paucity of data and knowledge gaps.

Fanconi anemia, cold agglutinin disease, mastocytosis, erythropoietic porphyria, and Castleman disease each had three cases with an incidence rate of 0.0066 and prevalence rates ranging from 0.084 to 0.126 per 100,000 adults. These conditions, while still rare [11,26-28] showed a small presence in the population.

Common variable immunodeficiency (CVID) is estimated to affect about one in 25,000 individuals, with a notably higher prevalence observed in Northern Europe and most commonly diagnosed after puberty. The majority of cases were identified in individuals aged between 20 and 45 years [29] and were identified once, with an incidence and prevalence rate mirroring those of other ultra-rare diseases in the study at 0.0022 and 0.042 per 100,000 adults, respectively.

Our study has significant implications for healthcare in Albania. It underscores the need for healthcare resource allocation, specialised care centres, and targeted therapies for patients with rare haematological diseases.

The higher prevalence of certain conditions in our study underscores the importance of maintaining disease registries and investing in epidemiological research. Maintaining disease registries is crucial for monitoring and understanding the epidemiology of rare diseases. Our study reinforces the value of these registries in capturing essential data for healthcare planning and research. International collaboration is vital for improving the knowledge and management of rare haematological diseases. Comparative studies with neighboring regions can provide insights into regional disparities in disease burden.

The comparison of our data with global statistics highlights the variability in disease prevalence and incidence, which could stem from differences in study methodologies, healthcare access, and genetic factors among populations. Consequently, our study contributes valuable data to the global rare disease registry.

## Conclusions

In conclusion, our study, conducted at the University Hospital Center "Mother Teresa" in the Hematology Service - the sole diagnostic center for blood diseases in Albania - makes a modest yet significant addition to the global registry of rare diseases. It highlights the critical importance of understanding rare hematological diseases within the Albanian context. Our research contributes valuable data to the scant literature on these diseases in Albania and emphasizes the need for region-specific studies. Such studies are vital in identifying unique epidemiological patterns, which can inform more effective healthcare strategies and resource allocation for the management of rare diseases in distinct geographical areas. Additionally, our findings underline the necessity of establishing a rare disease registry in Albania. This registry would not only enhance diagnostic capabilities but also improve the standard of patient care, addressing the unique challenges faced by individuals with these conditions.

Moreover, the data provided by our research lay the foundation for future studies and the formulation of healthcare policies aimed at improving the lives of those affected by rare hematological diseases in Albania. The impact of our work transcends national boundaries, contributing to the global discourse on rare diseases. We are optimistic that our research will pave the way for further advancements in this field, ultimately leading to better health outcomes and quality of life for individuals afflicted with rare hematological conditions.
